# Patient experiences of a lifestyle program for metabolic syndrome offered in family medicine clinics: a mixed methods study

**DOI:** 10.1186/s12875-018-0837-z

**Published:** 2018-08-31

**Authors:** Jennifer Klein, Paula Brauer, Dawna Royall, Maya Israeloff-Smith, Doug Klein, Angelo Tremblay, Rupinder Dhaliwal, Caroline Rheaume, David M. Mutch, Khursheed Jeejeebhoy

**Affiliations:** 1 Glenrose Rehabilitation Hospital, Edmonton, Canada; 2 Department of Family Relations & Applied Nutrition, University of Guelph, Guelph, Canada; 3 Department of Family Medicine, University of Alberta, Edmonton, Canada; 4 Department of Kinesiology, Laval University, Quebec City, Canada; 5 Metabolic Syndrome Canada, Kingston, Canada; 6 Department of Family Medicine and Emergency Medicine, Laval University, Quebec City, Canada; 7 Department of Human Health & Nutritional Sciences, University of Guelph, Guelph, Canada; 8 Department of Medicine, University of Toronto, Toronto, Canada

## Abstract

**Background:**

Patient perspectives on new programs to manage metabolic syndrome (MetS) are critical to evaluate for possible implementation in the primary healthcare system. Participants’ perspectives were sought for the Canadian Health Advanced by Nutrition and Graded Exercise (CHANGE) study, which enrolled 293 participants, and demonstrated 19% reversal of MetS after 1 year. The main purpose of this study was to examine participants’ perceptions of their experiences with the CHANGE program, enablers and barriers to change.

**Methods:**

A convergent parallel mixed methods design combined patients’ perspectives collected by questionnaires (*n* = 164), with insights from focus groups (*n* = 41) from three sites across Canada. Qualitative data were thematically analyzed using interpretative description. Insights were organized within a socio-ecologic framework.

**Results:**

Key aspects identified by participants included intra-individual factors (personal agency, increased time availability), inter-individual factors (trust, social aspects) and organizational factors (increased mental health support, tailored programs).

**Conclusion:**

Results revealed participants’ overall support for the CHANGE program, especially the importance of an extended program under the guidance of a family physician along with a skilled and supportive team. Team delivery of a lifestyle program in primary care or family medicine clinics is a complex intervention and use of a mixed methods design was helpful for exploring patient experiences and key issues on enablers and barriers to health behavior change.

**Electronic supplementary material:**

The online version of this article (10.1186/s12875-018-0837-z) contains supplementary material, which is available to authorized users.

## Background

In Canada, 19% of adults have metabolic syndrome (MetS) [1, 2] exhibiting at least three of five common risk factors: high waist circumference, increased blood pressure, elevated blood glucose, elevated triglycerides and decreased high-density cholesterol levels. People with MetS have been shown to have double the annual health care costs and higher frequency of services than those without MetS [3, 4]. Progression of MetS to diabetes and heart disease can be significantly reduced by changes in diet and exercise [5–8]. Most people with MetS are treated in the primary care system.

Building on promising results from previous studies [9–11], the Canadian Health Advanced by Nutrition and Graded Exercise (CHANGE) feasibility study was completed in three diverse primary care organizations across Canada to demonstrate the possibility of achieving reversal of MetS within the Canadian primary care context. [11] This program was a practical, flexible, and personalized diet-exercise program delivered by a team of health professionals (i.e., family physician, dietitian, and exercise specialist). The CHANGE intervention was driven by the ongoing relationship that patients have with their family doctor. Key features of the program included: 1) family physician encouragement and ongoing monitoring; 2) intensive diet and exercise with individualized counseling and support by dietitians and exercise specialists each week for 3 months; followed by 3) monthly visits for the remaining part of the 1 year period. The program was successful in reversing MetS among 19% of the 293 patients and details are described elsewhere [11, 12].

Evaluation of patients’ perspectives is particularly important for lifestyle programs, as programs vary widely and attrition can be substantial [13, 14]. Mixed methods study designs are becoming increasingly popular, being particularly helpful in identifying diverse issues and in engaging a larger cross-section of participants. The socio-ecologic framework, a theory-based framework for understanding the complex interplay of personal and environmental factors that determine behaviors, was used to organize insights at three levels: intra-individual, inter-individual, and primary care organization levels [15].

The main purpose of this study was to examine participants’ perceptions of their experiences with the CHANGE program, enablers and barriers to change. Participants were from all three sites (i.e., Edmonton, Toronto and Quebec City).

## Methods

A convergent parallel mixed methods design was used [16], with questionnaire and focus group data collection and analyses conducted concurrently. Results were analyzed separately and then considered together in an overall interpretation of the issues of interest. Written consent was obtained from the participants in advance of participation. Ethical approval was obtained from the University of Alberta Health Research Ethics Board.

### Questionnaire

#### Questionnaire development

The questionnaire was a tool for assessing patients’ experiences with lifestyle programs in primary care. It was developed through input from primary health care providers regarding the critical issues to be assessed for quality improvement purposes in primary care, based on a review of key indicators of primary care service quality [17]. Content validity was established through interviews with patients participating in a lifestyle program. It consisted of 21 multiple choice and 2 open-ended questions on the following dimensions as described by Wong and Haggerty [18]; access, interpersonal communication, continuity and coordination, comprehensiveness of services, trust, and patient-reported impacts of care. (See Additional file 1). The questionnaire was used as published [17], with the generic introduction modified to be specific to the CHANGE project and the entire questionnaire translated into French and Russian to meet the language needs of the participating sites.

#### Questionnaire administration, collection and analysis

Questionnaire administration and data collection was managed by research coordinators (RCs) from each of the three sites. Each RC had options for getting the questionnaires completed. They could complete with patients in-person or mail paper questionnaires in the languages appropriate for their site along with pre-stamped return envelopes. The French and English paper questionnaires also included a web address to the online questionnaire so that participants could complete the questionnaire online if they wished. The online questionnaire was not translated into Russian, as the RC at the site opted to translate and complete questionnaires with participants in-person. All paper questionnaires were transcribed and downloaded into Microsoft Excel for descriptive analysis. Comments written were content analyzed for themes by one researcher (DR) and reviewed by two members of the team for consensus on theme content (PB and DR).

### Focus groups

Focus group participants consisted of individuals currently participating in the CHANGE study and were recruited through a mail-out to all participants, outlining the purpose of the study. In addition, flyers were posted in the clinic, inviting people to participate in the study. Potential participants were asked to contact the researcher either by phone or email. Focus groups took place in all three cities. An experienced qualitative researcher experienced in facilitating focus groups (JK) established a relationship at the beginning of the sessions outlining the purpose of the research, her role in the project and then moderated the English sessions. The focus group in French was led by a francophone researcher (AT) who established a relationship at the beginning of the sessions with participants, outlining the purpose of the research, his role in the project and then facilitated the discussion, with input from the original facilitator (JK) who is bilingual, but had French as a second language. Open-ended questions were used to explore participant perspectives. (See Additional file 2.) Focus groups lasted approximately 1 hour each and were conducted at the participants’ respective health care centre. Field notes were made after the focus groups.

#### Focus group analysis

Discussions were digitally recorded, transcribed and organized using NVIVO software [19], a textual database computer software program. Transcripts were analyzed by an experienced qualitative researcher (JK) using Interpretive Description, a non-categorical approach to qualitative data analysis that goes beyond description of a phenomenon to a meaningful interpretation [20]. In keeping with the general principles of interpretive description methodology, the analytic process was inductive, using constant comparative analysis to track commonalities and variations among and between participants according to the research question. Data was organized and analyzed involving coding ideas and refining themes as patterns emerged [21]. Codes were designed to capture details surrounding the experiences of participating in the CHANGE program. These codes were then loosely organized into overarching 15 themes and subthemes (Additional file 3). These thematic patterns were identified and explored, allowing for minor adjustments in the probing questions for subsequent focus groups on issues where expansion and clarification seemed potentially fruitful. Member checking occurred at the final segment of the four focus groups to validate focus group themes; participants were provided with a summary of themes gathered to that point in the research project. Participants were asked to review and provide comments, serving as a check on the viability of the interpretations.

### Availability of data and materials

The questionnaire is available in Additional file 1. Focus group questions are available in Additional file 2. Examples of themes and subthemes for focus group analysis are available in Additional file 3. Datasets used and analyzed during the current study are available from the corresponding author on request.

## Results

### Questionnaire

A total of 164 questionnaires were received (57 French, 107 English and 0 Russian) of 293 potential recipients (56% response rate); 76% as paper questionnaires and 24% online responses. Of the 164 responses to the survey, 27 (19.4%) respondents reversed their metabolic syndrome. This is consistent with the 19% reversal found in the entire study population (11). Therefore, the survey response reflects the population included in the larger study. Most questionnaire respondents (89%) had completed the one-year program; thus, results mostly reflect their perspectives. Numbers were inadequate to conduct subgroup analysis on the drop-outs.

#### Program enablers

**Fig. 1:**
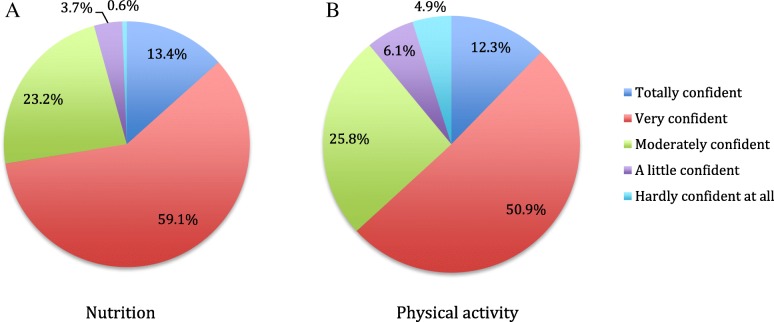
Participants’ ratings of confidence in maintaining changes in nutrition and physical activity. In response to the questions: A. How confident are you that you can maintain the changes in your nutrition ( *n* = 164); B. How confident are you that you can maintain the changes in your physical activity ( *n* = 163). *p* < 0.05 by Chi-square test

Results of the quantitative aspects of the questionnaire and the dimension being assessed are shown in Table 1. With respect to interpersonal communication, virtually all patients (99%) felt respected and program’s elements were clearly explained. Trust in the providers and their information was also very high. Participants felt they were rarely or never told different things by different providers (team functioning). While only 76% felt they were definitely involved in setting their goals, 88% felt their personal situation was taken into account when providers made recommendations. During the program, 81% felt they had received the information they needed to make changes, with 17% indicating some gaps. On a 10-point scale, 80% rated the program 8–10 regarding the importance of the lifestyle program to their health, with another 17% rating at 5–7. A notable result was the finding that 71% thought the one-year program was the right length, while 19% found it too short. Even after 1 year, confidence in maintaining nutrition and physical activity changes was generally high, with the majority being at least moderately confident that they could maintain the diet and exercise changes (see Fig. 1).

**Table 1:** Responses to survey questions based on key dimensions assessed

Dimension (sub-dimension)	Question	*N* (%)
Access (First contact accessibility)	Able to contact a team member:	
	• Yes, definitely	125 (76%)
	• Yes, to some extent	14 (9%)
	• No, not at all	1 (1%)
	• Did not need to contact	24 (15%)
Access (Economic accessibility)	Difficulty in getting to appointments due to costs or time	
	• Never/Rarely	112 (68%)
	• Sometimes	42 (26%)
	• Often/Very often	10 (6%)
Interpersonal communication (General communication)	Knowledge of what to expect re: number of appointments, what would be learned and amount of support	
	• Yes, definitely	98 (62%)
	• Yes, to some extent	50 (32%)
	• No, not at all	10 (6%)
Interpersonal communication (General communication)	How often team members explained things clearly	
	• Always/usually	160 (98%)
	• Sometimes	2 (1%)
	• Rarely/never	1 (0.6%)
Interpersonal communication (Respectfulness)	How often team members treated with courtesy and respect	
	• Always/usually	163 (99%)
	• Sometimes	0
	• Rarely/never	1 (0.6%)
Interpersonal communication (Shared decision-making)	Involvement in setting goals	
	• Yes, definitely	122 (76%)
	• Yes, to some extent	37 (23%)
	• No, not at all	2 (1%)
Interpersonal communication (Whole-person care)	Did team members consider personal situation when making recommendations?	
	• Yes, definitely	143 (88%)
	• Yes, to some extent	19 (12%)
	• No, not at all	0
	• Don’t know	1 (0.6%)
Continuity and coordination (Team functioning)	How often told different things by different providers	
	• Always/usually	7 (4%)
	• Sometimes	10 (6%)
	• Rarely/never	144 (89%)
Comprehensiveness of services (Services provided)	How would you rate importance of this service to your health? (0 = not important at all; 10 = extremely important)	
	• 1–4	5 (3%)
	• 5–7	28 (17%)
	• 8–10	128 (80%)
Comprehensiveness of services (Services provided)	Appropriateness of program length?	
	• Too short	31 (19%)
	• Just right	114 (71%)
	• Too long	15 (9%)
Comprehensiveness of services (Services provided)	Have the team members provided the information and support needed to make changes?	
	• Yes, definitely	132 (81%)
	• Yes, to some extent	28 (17%)
	• No, not really	2 (1%)
	• Not needed	1 (0.6%)
Trust	Comfortable sharing personal information with team?	
	• Yes, definitely	152 (93%)
	• Yes, to some extent	12 (7%)
	• No, not at all	0
Trust	Confidence in information received	
	• Yes, definitely	153 (93%)
	• Yes, to some extent	11 (7%)
	• No, not at all	0
Patient-reported impacts on care (Action on Goals)	Confidence in maintaining changes in nutrition	
	• Totally/very confident	119 (73%)
	• Moderately confident	38 (23%)
	• Little/hardly confident	7 (4%)
Patient-reported impacts on care (Action on Goals)	Confidence in maintaining changes in physical activity	
	• Totally/very confident	103 (63%)
	• Moderately confident	42 (26%)
	• Little/hardly confident	18 (11%)
Patient-reported impacts on care (Action on Goals)	Were there times when did not try nutrition changes because something got in the way?	
	• No	58 (37%)
	• Yes, sometimes	89 (57%)
	• Yes, often	10 (6%)
Patient-reported impacts on care (Action on Goals)	Were there times when did not try physical activity changes because something got in the way?	
	• No	67 (44%)
	• Yes, sometimes	75 (49%)
	• Yes, often	11 (7%)

Over 150 positive qualitative comments from the questionnaire, representing more than 80% of the comments, indicated patients felt the personalized program was motivating and had increased their awareness and knowledge of what to eat and how to exercise. Increased self-efficacy was often mentioned such as: “*They provided me with the confidence and knowledge to succeed”*. Further, patients commented that their behavior, health, and well-being had improved *(*e.g.*, “not as susceptible to fatigue”* and *“I feel less like snacking during the day or the evening”)*. Several patients noted reduced medication use or decreased blood pressure.

#### Program barriers

Some challenges with the program emerged. For example, 32% indicated they ‘sometimes’ or ‘often’ had problems getting to appointments due to time or cost. Only 62% felt they knew what to expect at the start of the program and 23% felt they had been involved in setting goals only to some extent.

Many participants (i.e., 49–57%) reported sometimes not trying the recommended diet or activity changes because something got in the way, while only 6–7% reported that this happened often (see Table 1). Qualitative comments denoting barriers focused on challenges maintaining the changes, such as work schedules and winter weather. Other key barriers for diet changes included the need for fast and easy food preparation, as well as the role and emphasis of food in socializing and special occasions. Others reported challenges included adhering to a specific diet pattern, *“It’s hard to stick to a Mediterranean diet when you live in Alberta”*. The most common barrier to physical activity was chronic pain due to arthritis and other health issues.

When asked about services that should be added, removed or changed, a wide variety of suggestions emerged. Many participants felt the program could have been longer or more intensive with an increased frequency of meetings after the weekly phase was completed, and/or with more options for follow-up with providers or other participants. Several requested more evening and weekend appointments. Other suggestions included: encouraging participation of partners and household members, providing specialized support for mental health issues, and tailoring physical activities to participants’ differing abilities, interests and pain issues.

### Focus groups

There were 41 participants (15 females, 26 males) who participated in 6 focus groups across 3 clinics, each consisting of 6–10 participants. The focus group participants reflected the larger study population, including mean age of 60 (range 45–68 years), mean BMI 32, majority Caucasian along with some participants of Asian, Russian and Arabic ethnicity.

#### Overall experiences

Similar to the questionnaire, focus group participants reported very positive feedback about being a part of the CHANGE program. Many shared they felt it was *“a privilege”* to be part of the program and reported the program had changed their life by educating, supporting, and implementing realistic lifestyle changes.

#### Program enablers

Individualized gradual approach to care. Participants emphasized the personalized and gradual approach was the biggest asset of the program and helped to differentiate it from other diets and programs previously tried as it was realistic and conceivable to adapt to individual needs. The program was reported to be *“structured, yet flexible”* enough to meet a variety of dietary, exercise, and lifestyle needs.

“It’s a very structured approach to changing your diet and exercising…yet it’s so flexible. The diet is about what I wanna eat and not about what somebody else is shoving down your throat.” (Participant 12)

Personalities and approach of the staff. The support and enthusiasm of the staff were key factors in the adherence and success of participants. Staff reportedly created a relaxed atmosphere, never making participants feels rushed, yet “*providing gentle discipline…without being viewed as pushy*”. Many comments were made that staff were not judgmental. “*They didn’t sit there and judge you, and say ‘oh you shouldn’t be drinking all that wine or having all that red meat…I know I was doing things wrong and I think we all know we have to make changes in our lives’*”.

Physicians as motivator and trusted mentor. Physicians played an important role in participants registering for the program. Several participants said they would not have registered independently, but did so on recommendation from their physician. Participants also spoke of adhering to the program, as they felt accountable to their physicians.

“I think if the doctor is suggesting it, that probably was one of the most powerful incentives.” (Participant 27).

Frequency of meetings for the program. Surprisingly the intense weekly visits for the first 3 months were not seen as a barrier and were very well received. Not one participant felt it was too much. “*The first three month period is very good. Reinforces everything. Kind of kick starts you*.”

Accessible supervision by expert team. Participants appreciated that there were expert health professionals to guide them. Several mentioned they were apprehensive to try new lifestyle changes due to their lack of knowledge in the realms of healthy eating and exercises and/or implications of lifestyle changes on their illness or recent surgeries. Having the team involved provided confidence for them to implement the changes. Participants appreciated the open access to the dietitians and kinesiologists at all times.

Social aspect. The social aspect of the program was prominent.

“I can say coming to exercise in a group helped me because I think when you just do it by yourself or with your instructor you don’t feel as much part of something that’s going on, so it was always kind of neat to come to the gym and see other people that were on treadmills… That were all at different stages… that helped because I think I had felt for a long time there was no way I was ever gonna get any success out of weight loss program.” (Participant 3).

Impact on significant others. Participants spoke of the impact of the program was not just individual. “*My wife got on board so that it became a family-oriented thing rather then just an individual thing*”. Participants spoke of the program influencing family members and friends, which was a great motivator.

“I shared information with two colleagues. There were family members who wanted to ride with me, who wanted to exercise. It’s inspiring to them. There is an impact on the family. The meals are no longer the same. There are no hamburgers and fries anymore…people around me also changed their habits. You can change the habits of 10 people through only one person.” (Participant 12).

#### Program barriers

While participants were very positive about their experiences, several challenges were shared. These were divided into personal and program-based.

Personal. The biggest challenge was the psychological barrier to changing personal habits and maintaining self- discipline. As one participant declared, “*Controlling your diet is harder than quitting smoking*”. Several participants spoke of the difficulties of maintaining their health goals when family members were not supportive. “*My biggest challenge has been trying to make the food changes with the rest of my family, who are not in the CHANGE program, and don’t want to change*”. Similar to the questionnaire findings, many participants shared that the lack of time to participate in exercise was challenging.

Program. There was a minimal amount of support to address mental health issues. Participants mentioned there had been a small assessment with the nutritionist, but would prefer a larger focus in this area. Offering access to a mental health professional early on in the program was deemed helpful.

“Those services take a long time to get. The assessment was done and the expectation was that your family doctor followed up on that but who do family doctors refer to? Psychologists, psychiatrists, so you’re just putting another stumbling block of time and energy. Here I am on month 6, finally mentally in the game. The year’s half done.” (Participant 34).

In addition, several participants suggested more variety in the exercise programs and increased focus on incorporating specific health issues (e.g., arthritic knees). The program offered appointments and services only during the weekdays. Several participants requested evenings and weekend access due to conflicts with work schedules.

## Discussion

Despite the questionnaire and focus group questions being framed differently, similar issues emerged from participant’s responses. The focus groups elicited more reflection on intra-personal issues, while organizational issues were more prominent in the questionnaire results.

### Intrapersonal level

Published literature reports the most prevalent and important barriers to lifestyle change, from participants’ perspectives, are ‘lack of willpower’, self-discipline, and personal agency or self-efficacy, depending on the theoretical model being used [22, 23]. Lack of time (due to family, household and occupational responsibilities) is also a significant barrier that has been extensively discussed in the health behavior change literature [24–26].

In the current study, changing habits was personally challenging, but participants shared that the program mitigated some of the challenges listed above. Comments on the individualized aspects of the program, the supportive and knowledgeable personalities of the providers and the social aspects were all seen as important to promote ongoing motivation and participation.

Results from both the questionnaire and focus groups reinforced the well-known challenges with maintaining changes, even after a one-year program. While over 60% were totally or very confident to maintain both diet and physical activity changes, there was a subgroup of about 10% who were less confident in maintaining physical activity changes and 4% for dietary changes. Optimal program length for the majority of people to maintain new lifestyle habits remains controversial and has implications for program delivery costs and sustainability.

### Interpersonal level

Trust has been found to be an important element to consider in patient assessment of services [27]. Focus group results highlighted the importance of the family physician as motivating the participants to change, while also acknowledging the skills and knowledge of the other providers. The role of the family physician as a trusted mentor is likely critical to the success of the program.

While the family physician plays a key role as a coordinator and mentor, team functioning is also known to be very important in delivery of complex interventions [28]. This was not directly assessed, as patients might not know how teams work. It was indirectly assessed in the questionnaire by asking if patients received contradictory information on diet and exercise. Most felt that different providers did not tell them different information, an issue that has been identified in some team programs [29].

People enjoyed socializing with other participants during the program. While most health counselors are trained on motivational interviewing and other counseling techniques, they may not understand the importance of, nor have been formally trained on the social benefits of the program. Since, providers in this study usually counseled one-on-one, the social aspects of the program should be further enhanced. The importance of having significant others as part of the program was mentioned both in the questionnaire comments and in the focus groups. Greater appreciation for health behavior change influencing the whole household has implications for further development of the CHANGE program.

### Organizational level

Mental health is a significant factor impacting behavior change programs [30]. In both the questionnaire and focus groups, participants reported that better mental health resources and supports were needed to provide increased success in the program. Feedback from participants highlights the importance of providing focused mental health related to low mood, fears, negative perceptions of health and life changes, and lack of motivation. Surprisingly, no studies were found that examined the impact of providing an experienced mental health worker in a similar lifestyle change program for MetS. However, the importance of providing psychological supports is reinforced in other studies [30–33].

Several comments support a need to tailor the CHANGE program to various subgroups. Many middle-aged participants were still in the workforce, so time limitation was a key barrier. Offering more evenings and weekend programming would be beneficial for this group. At the organizational level, patients often advocate for increased flexibility regarding accessing services including increased flexible clinic hours. Through the development of primary care reform, clinics are starting to address this need by hosting clinics on evenings and weekends. However, allied health services need to be expanded as well. The older adult population had more flexibility in their time, but had higher levels of social disconnectedness and perceived isolation, which are independently associated with lower levels of self-rated physical health [34]. Seniors also encounter more barriers e.g., (transportation, increased physical activity limitations). Participants with lower socioeconomic status also need to be considered as they too have increased barriers (e.g., cost of parking, public transportation, child care costs, absence from work to attend appointments) as well as low participation rates in lifestyle change programs [35, 36]. Consideration of reimbursement for out-of-pocket expenses related to participating in the program could assist with attracting more participants from this subgroup. As some cultural minorities experience a high prevalence of MetS and engage in leisure time physical activities less frequently than do adults in the rest of the population [37], developing strategies to encourage this group to participate in CHANGE would also be important. For example, targeting the program toward participants with similar cultural and linguistic backgrounds, recruiting health professionals who speak the language, and weaving components of the culture such a traditional dance and ethnic diets into the program. Further development will need to address differing issues in differing locations.

Themes from the focus groups frequently complimented the questionnaire. There were no direct contradictions in comparing the results of questionnaire and focus groups; rather different aspects emerged as important. The key role of the family physician and importance of the social aspects emerged in the focus groups, while the questionnaire confirmed the trust they had in the team. Both methods revealed trust with their health professionals, along with an individualized realistic gradual approach led to the general support for such a lengthy and intensive program. Findings that a longer and more intensive program was preferred were unexpected and needs to be further explored.

### Limitations

Further validation of the questionnaire is needed including assessment of test-retest reliability and convergent and divergent validity. While we sent out the questionnaire to all participants in the study, the response rate of 56% is likely not representative. In addition, as the focus group consisted of participants self –selecting and were only offered in French or English, not Russian, there is potential for further bias. Extra concerted efforts would be needed to elicit perspectives from participants, who declined to participate in the questionnaire and focus groups, which was beyond study scope. We also have no knowledge of the needs of the wider group of people with MetS who did not agree to undertake the CHANGE intervention. Different approaches are needed to determine how to reach and promote lifestyle change among other subgroups in the population. Likely multiple strategies are needed, including web-based approaches, group programs, and programs with a stronger mental health focus. Further work is required to explore the 10% of participants who noted they were less confident in maintaining physical activity changes and 4% for dietary changes.

## Conclusion

This research provides insight into patients sharing their experience of the CHANGE lifestyle program in family medicine clinics and generally confirms a large body of literature on the major enablers and barriers to lifestyle change. Overall participants’ experiences were very positive especially as they emphasized the importance of participating in an individualized program led by family physicians and a skilled and supportive team. The role of the family physician as a trusted mentor is likely a key factor to the success of the program.

Due to the personalized supports, participants’ confidence in maintaining the changes long term was high. Specific recommendations provided by participants (e.g., increased mental heath support, flexible hours, tailoring program to specific diseases, including families in the program) may further improve the CHANGE program and its long-term success and sustainability.

### Practice implications

Implementing complex behavioral interventions such as lifestyle modification in primary care is challenging. Although intrapersonal issues (e.g., intention, self-efficacy, self-discipline) often dominate peoples’ perceptions of the success of such an intervention, modifying aspects at the interpersonal (e.g., trust, team functioning) and organizational level (e.g., mental health supports, flexible hours) can ultimately improve the effectiveness of a program. The positive participant experiences shown in our current work enhance the evidentiary basis of the CHANGE Program and supports the wide spread implementation of CHANGE in primary care. Multiple approaches may be needed to obtain future participant experiences to inform ongoing program improvements.

## Additional files

Additional file 1: Survey questionnaire. (DOCX 228 kb)

Additional file 2: Focus Group questions. (DOCX 13 kb)

Additional file 3: Examples of Emergent themes and sub-themes based on analysis of focus groups. (DOCX 15 kb)

## Abbreviations

CHANGE: Canadian Health Advanced by Nutrition and Graded Exercise
MetS: Metabolic syndrome
