# Supplementary material for: Patient experiences of a lifestyle program for metabolic syndrome offered in family medicine clinics: a mixed methods study
Source: BMC Fam Pract. 2018 Aug 31;19:148. doi: 10.1186/s12875-018-0837-z (PMC6119314; doi:10.1186/s12875-018-0837-z)
Supplement: Supplementary file 1 — Survey questionnaire. (DOCX 228 kb) [file 12875_2018_837_MOESM1_ESM.docx]

**Additional file 1 – Survey questionnaire**

**
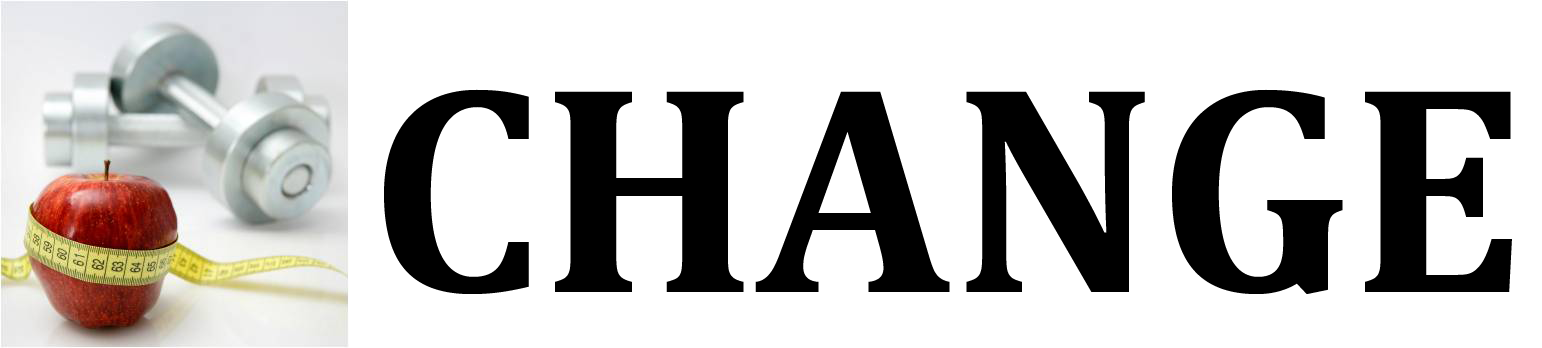
**

🗆🗆 🗆 🗆🗆🗆

Site # Protocol Inscription #

Patient Exit Questionnaire

Canadian Health Advanced through Nutrition and Graded Exercise (CHANGE) study

Please complete this survey and send back by mail using the stamped, addressed envelope to:

Dr. Paula Brauer

Dept Family Relations and Applied Nutrition

50 Stone Road East

Guelph, ON N1G 2W1

OR you may also complete online at:

<http://bit.ly/1tINEY7>

The CHANGE study has been an individualized diet and exercise program offered through your doctor’s office. We are interested in your experiences with the CHANGE program so we can expand and improve services for others.

You are being invited to take part in this survey because you have met with one or more team members through the CHANGE study. Team members may include the dietitian, exercise specialist, doctor, nurse, or other people working in the clinic.

Before you answer, please remember that:

- You can choose whether to fill in the survey or not.
- You can skip any question you are not comfortable answering.
- No one will know who answered this survey.
- There are no right or wrong answers.

All questions are about the CHANGE program.

1. How did you find out about this service?

□ A doctor, nurse practitioner, nurse told me

□ Another healthcare professional told me

□ Advertised in the office

□ A friend told me

□ Other: _____________________________

1. How did you access this service?

□ Referred by doctor, nurse practitioner, or nurse

□ Referred by other healthcare professional: _______________________

□ I referred myself

□ Other:______________________________

1. How would you rate the importance of this service to your health? Please circle.

(0 = not important at all, 10 = extremely important)

| 0 | 1 | 2 | 3 | 4 | 5 | 6 | 7 | 8 | 9 | 10 |
| --- | --- | --- | --- | --- | --- | --- | --- | --- | --- | --- |

1. How long did you wait from the time you were referred to the time you had your first appointment?

□ Less than 2 weeks

□ 2 to 4 weeks

□ 5 to 8 weeks

□ More than 8 weeks

□ I don’t know

1. Did you stop attending before the program finished?

□ No

□ Yes

If Yes, please share what influenced you to stop attending appointments before the program finished.

1. Did you know what to expect from the nutrition and/or physical activity services in terms of how many appointments you would have, what you would learn, and the amount of support you would be offered?

□ Yes, definitely

□ Yes, to some extent

□ No, not at all

Comments:

1. How would you describe the program length to support your lifestyle changes?

□ Too short

□ Just right

□ Too long

1. Were there times when you found it difficult to get to nutrition and/or physical activity appointments because of the additional costs or time it involved (e.g. childcare, parking, transportation, work schedule, other commitments)?

□ Never

□ Rarely

□ Sometimes

□ Often

□ Very often

Comments:

Questions 9-16 relate to ALL the different people (team members) that you saw through this program to support nutrition and/or physical activity. Please add any comments you have for the questions below:

1. Were you able to contact a team member about your nutrition and/or physical activity when you wanted to?

□ Yes, definitely

□ Yes, to some extent

□ No, not at all

□ I did not need to contact a team member

1. How often did the team members explain things to you in a way that you clearly understood?

□ Always

□ Usually

□ Sometimes

□ Rarely

□ Never

1. Were you comfortable sharing personal information with the team members?

□ Yes, definitely

□ Yes, to some extent

□ No, not at all

1. How often did the team members treat you with courtesy and respect?

□ Always

□ Usually

□ Sometimes

□ Rarely

□ Never

1. Did the team members consider your personal situation (lifestyle, income, traditions, and culture) when making nutrition and/or physical activity recommendations for you?

□ Yes, definitely

□ Yes, to some extent

□ No, not at all

1. Did you have confidence in the information about nutrition and/or physical activity that the team members gave you?

□ Yes, definitely

□ Yes, to some extent

□ No, not at all

1. How often were you told different things (that didn't make sense together) about nutrition and/or physical activity?

□ Always

□ Usually

□ Sometimes

□ Rarely

□ Never

1. Have the team members provided the information and support you need to make changes to your nutrition and/or physical activity?

□ Yes, definitely

□ Yes, to some extent

□ No, not really

□ No, not at all

□ No, I have not needed information and support

Please add any comments that relate to ALL the different people (team members) that you saw through this program to support nutrition and/or physical activity

1. Were you involved in setting nutrition and/or physical activity goals to address the health issues that matter to you?

□ Yes, definitely

□ Yes, to some extent

□ No, not at all

1. How confident are you that you can maintain the changes in your nutrition?

□ Totally confident

□ Very confident

□ Moderately confident

□ A little confident

□ Hardly confident at all

1. How confident are you that you can maintain the changes in your physical activity?

□ Totally confident

□ Very confident

□ Moderately confident

□ A little confident

□ Hardly confident at all

1. Were there times when you did not try the suggested nutrition changes because something got in the way?

□ No

□ Yes, sometimes

□ Yes, often

If yes, please share what you experienced:

1. Were there times when you did not try the suggested physical activity changes because something got in the way?

□ No

□ Yes, sometimes

□ Yes, often

If yes, please share what you experienced:

1. How has working with the team members on nutrition and/or physical activity helped you better manage your health?
2. Are there services that you think should be added, removed, or changed?

Thank you! Your feedback is important.

It will be used to make improvements to services.
