# Supplementary material for: Patient experiences of a lifestyle program for metabolic syndrome offered in family medicine clinics: a mixed methods study
Source: BMC Fam Pract. 2018 Aug 31;19:148. doi: 10.1186/s12875-018-0837-z (PMC6119314; doi:10.1186/s12875-018-0837-z)
Supplement: Supplementary file 2 — Focus Group questions. (DOCX 13 kb) [file 12875_2018_837_MOESM2_ESM.docx]

**Additional file 2**

**Focus Group questions**

Tell us about your experience with the CHANGE program.

What are the benefits/positive aspects for you to take part in this program?

What are some of the barriers/challenges to taking part in this program?

What were some of the most useful/least useful aspects of the program?

What types of support or assistance could be put in place to help you continue an exercise program?
