# Supplementary material for: Patient experiences of a lifestyle program for metabolic syndrome offered in family medicine clinics: a mixed methods study
Source: BMC Fam Pract. 2018 Aug 31;19:148. doi: 10.1186/s12875-018-0837-z (PMC6119314; doi:10.1186/s12875-018-0837-z)
Supplement: Supplementary file 3 — Examples of Emergent themes and sub-themes based on analysis of focus groups. (DOCX 15 kb) [file 12875_2018_837_MOESM3_ESM.docx]

**Examples of Emergent themes and sub-themes based on analysis of focus groups**

General experiences participating in CHANGE program

- Positive aspects: It’s a good program. It’s the exercise and the diet. The dietician seems to wanna know what you have from the time you get up till the time you go to bed, what you have to eat sort of thing. She’s very concerned, so it’s a good program
- Negative aspects: But for people that are retired they say that they got more time on their hands and they have money. The younger people to attend this… their employer wouldn’t appreciate them taking time off once a week to go to this program, whether it helps their health or not.

Unique program influencers

- Individualized care: I found the program to be very good, I’ve been to several other similar type studies or classes with 8 to 10 people, but on the one-to-one basis I found it very interesting. I can bring my ideas out or they can make a suggestion to me personally, that’s what I found about this program so far.
- Impact of physician involvement: I’d be willing to bet the way we all got into the program is a pretty strong hint, I think what you need to do to get other people in there is push GPs to get their patients into it. Because I’m thinking of a sister of mine, well she can’t get into this program ’cause she lives out at rurally and she’s a cattle rancher. If she had access to something like this I would be willing to bet that her doctor pushing her into it might get her in, but she’s just as pig headed as me.
- Impact of significant others: I can say that my husband’s not a demonstrative person, I can’t say he’s ever actually opened his mouth and said, gee this is great, or I’m glad you’re doing this, but he drives me all the time, and he sits outside and waits for 2 hours. So there’s ways that they show you, I figure he’s OK with it.
- Impact of electronics: I think tracking the pedometer helped. So it gave me an idea how many steps I was doing. Lots in the summer months, but at the beginning I was doing genealogy research so a lot on the computer, so then I realized I have not been out. So that has helped, the awareness of it.

Motivators

- Internal : It was the determination and the commitment that I stuck to and basically the weight I lost and the concern for everybody as to how you were doing and ideas like that.
- External : The tracking of the food, writing down what you eat, I think it would be hard to overstate the importance of that. It was really important for me just to know that the dietician is interested in you. I think that’s a powerful tool to write everything down/

Timelines: I found it difficult that after three months, from once a week, we went to once a month. I would have liked maybe every two weeks for 2-3 months before going once a month. The cut once a week to once a month, it's more difficult.

Outcomes: Well I lost 2 or 3 dress sizes, that was an early motivation. It’s wonderful to walk into a store and say OK, I was say a size 16, I’m going to try a 14, and then suddenly you get, and it’s not suddenly, it’s slowly, but it’s really wonderful when it comes off, and without becoming twiggy you do become more aware that you look nicer. Which I think is important that you just feel better about yourself, so that was a real treat.

Feedback for individual sites: Yes, if we could come to exercise when the kinesioloigst is there. This would have allowed me to meet the other participants, to mobilize all together. It would have been nice for the other participants, to see people coming and who have completed the program. This would have allowed exchange between participants.

Recommendations: Cooking classes would be helpful. I got to this point personally because I had no cooking skills growing up. I did participate in one of the classes that were offered here through the PCN and it was good but they’re not very often. They’re hard to get into, you have to sell a kidney to get into them so I wish there was a cooking component. Teach me how to use a lentil, I don’t know how use a lentil. I finally discovered 2 weeks ago how to use lentils.
